# *MDM4* rs4245739 A > C polymorphism correlates with reduced overall cancer risk in a meta-analysis of 69477 subjects

**DOI:** 10.18632/oncotarget.12326

**Published:** 2016-09-28

**Authors:** Chaoyi Xu, Jinhong Zhu, Wen Fu, Zongwen Liang, Shujie Song, Yuan Zhao, Lihua Lyu, Anqi Zhang, Jing He, Ping Duan

**Affiliations:** ^1^ Department of Obstetrics and Gynecology, The Second Affiliated Hospital and Yuying Children's Hospital, Wenzhou Medical University, Wenzhou 325027, Zhejiang, China; ^2^ Department of Pediatric Surgery, Guangzhou Institute of Pediatrics, Guangzhou Women and Children's Medical Center, Guangzhou Medical University, Guangzhou 510623, Guangdong, China; ^3^ Molecular Epidemiology Laboratory and Department of Laboratory Medicine, Harbin Medical University Cancer Hospital, Harbin 150040, Heilongjiang, China; ^4^ Zhejiang Provincial Key Laboratory of Medical Genetics, Wenzhou Medical University, Wenzhou 325035, Zhejiang, China

**Keywords:** MDM4, polymorphism, cancer susceptibility, meta-analysis

## Abstract

Mouse double minute 4 (MDM4) is a p53-interacting oncoprotein that plays an important role in the p53 tumor suppressor pathway. The common rs4245739 A > C polymorphism creates a miR-191 binding site in the *MDM4* gene transcript. Numerous studies have investigated the association between this *MDM4* polymorphism and cancer risk, but have failed to reach a definitive conclusion. To address this issue, we conducted a meta-analysis by selecting eligible studies from MEDLINE, EMBASE, and Chinese Biomedical databases. Odds ratios (ORs) and 95% confidence intervals (CIs) were used to assess the strength of the associations. We also performed genotype-based mRNA expression analysis using data from 270 individuals retrieved from public datasets. A total of 15 studies with 19796 cases and 49681 controls were included in the final meta-analysis. The pooled results revealed that the *MDM4* rs4245739C allele is associated with a decreased cancer risk in the heterozygous (AC vs. AA: OR = 0.82, 95% CI = 0.73−0.93), dominant (AC/CC vs. AA: OR = 0.82, 95% CI = 0.72−0.93), and allele contrast models (C vs. A: OR = 0.84, 95% CI = 0.76−0.94). The association was more prominent in Asians and population-based studies. We also found that the rs4245739C allele was associated with decreased *MDM4* mRNA expression, especially for Caucasians. Thus the *MDM4* rs4245739 A > C polymorphism appears to be associated with decreased cancer risk. These findings would be strengthened by new studies with larger sample sizes and encompassing additional ethnicities.

## INTRODUCTION

Based on the latest GLOBOCAN estimates, there were approximately 14.1 million new cancer cases and 8.2 million cancer-related deaths worldwide in 2012 [1]. Developing countries accounted for almost 57% of new cancer cases and 65% of cancer-related deaths [1]. According to the trend in cancer incidence, the expected number of new cancer cases will reach 22.2 million worldwide in 2030 [2]. Leading risk factors for cancer development include tobacco use, overweight/obesity, physical inactivity, and infection [1]. Moreover, molecular epidemiological studies have demonstrated that genetic factors including single nucleotide polymorphisms (SNPs), may also play an important role in carcinogenesis [3–9].

As the gatekeeper for cellular growth and division, the tumor suppressor protein p53 maintains genomic stability and regulating cell growth, division, and apoptosis. Dysfunctional p53 protein can lead to the initiation and progression of tumors [10]. Mouse double minute 4 (MDM4) protein is a structural homolog of MDM2, which contains a p53 binding domain at the N-terminus and a RING finger domain at the C-terminus. MDM4 has been shown to inhibit p53 transcriptional activity directly by binding to its transcriptional activation domain. Overactive MDM4 reduces p53 tumor suppression function and contributes to tumor formation and progression [11]. The MDM4 protein can also inhibit the degradation of MDM2 by interacting with its RING finger domain [11]. Overexpression of MDM4 is associated with tumor progression and poor prognosis [12–14]. Previous molecular epidemiology studies suggest that genetic variations in *MDM4* gene are associated with risk of various types of cancer [15–20].

Among the many *MDM4* polymorphisms, a common genetic variant rs4245739 A > C has been widely investigated for its association with cancer susceptibility [21–28]. This polymorphism is located in the 3′ untranslated region (UTR) of the *MDM4* gene, and creates a miR-191 target site that can lead to decreased expression of MDM4. However, the studies have generated controversial results regarding the association between this polymorphism and cancer risk. The possible reasons for the inconsistencies include differences in ethnicity and geographic location, as well as the limited sample size. To date, no meta-analysis has been conducted to comprehensively investigate the association of *MDM4* rs4245739 A > C with overall cancer risk. To address the controversy regarding this association, we performed the current meta-analysis to precisely define the effect of *MDM4* rs4245739 A > C polymorphism on overall cancer risk.

## RESULTS

### Characteristics of eligible studies

**Figure 1:**
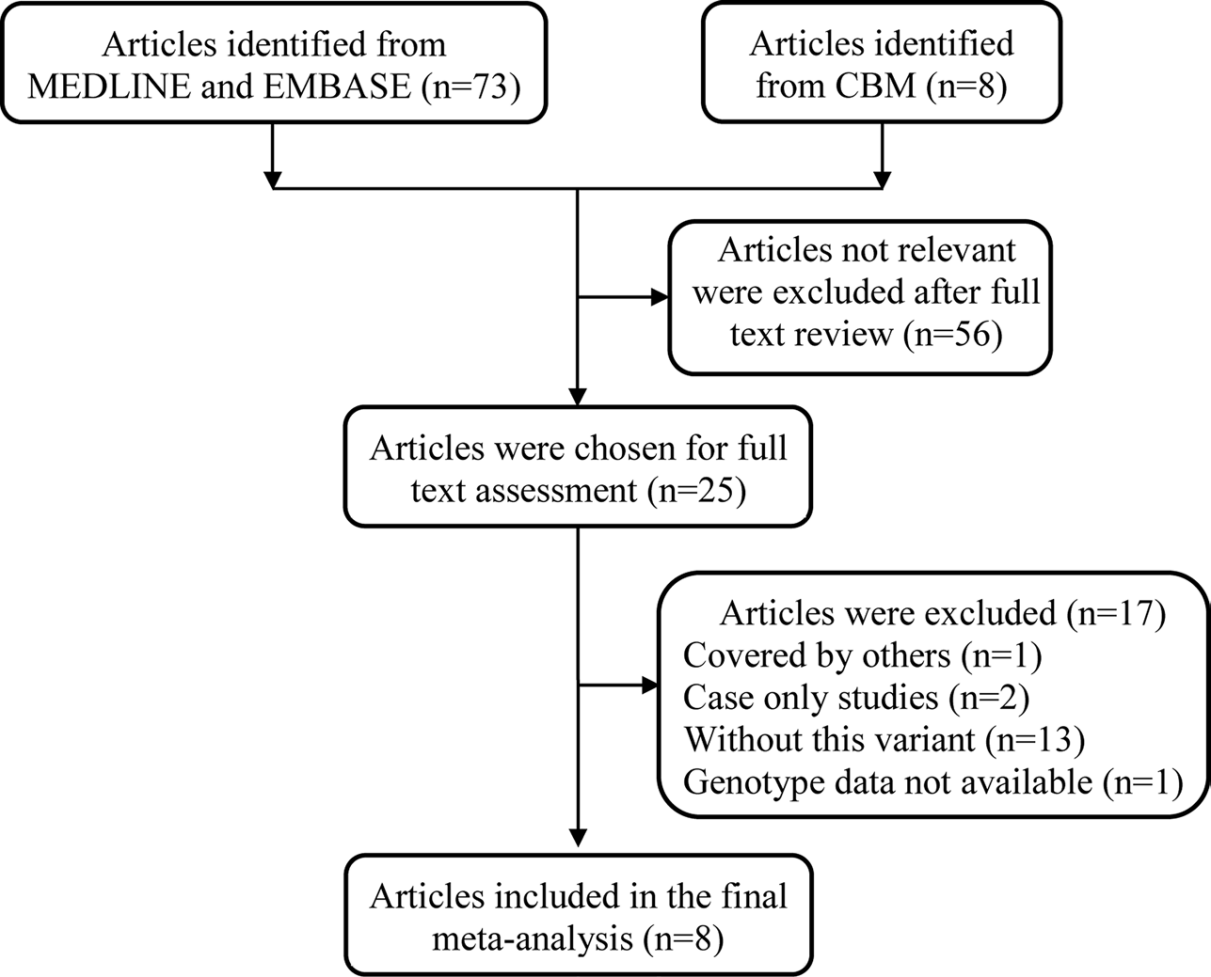
Flow diagram of included studies for the association between *MDM4* rs4245739 A > C polymorphism and overall cancer risk

A total of 81 articles were retrieved after an initial literature search in MEDLINE, EMBASE, and Chinese Biomedical (CBM) databases (Figure 1). After full text review, 73 articles were excluded for the following reasons: review articles, duplicate studies, non-case-control study design, genotype distributions were not available, or no evaluation of the association between *MDM4* rs4245739 A > C polymorphism and cancer risk. Ultimately, we found that only eight articles [21–28] met the inclusion criteria (Table 1). Among the eight articles, several investigations involving subjects from different areas were divided by area [22, 23, 27] and investigations were also separated by cancer type [26, 28]. As a result, a total of 15 case-control studies with 19796 cases and 49681 controls were included in the final meta-analysis. Of these, sample sizes ranged from 200 to 6512 for cases, and from 400 to 41451 for controls. The genotype distributions of the *MDM4* rs4245739 A > C polymorphism were in accordance with Hardy-Weinberg equilibrium (HWE) in the controls in all 15 studies. Studies were performed on various types of cancer. Four studies focused on breast cancer [21, 22, 26], three on lung cancer [26, 27], two on esophageal squamous cell carcinoma [23], and one each on non-Hodgkin lymphoma [24], gastric cancer [25], colon cancer [26], prostate cancer [26], ovarian cancer [28] and endometrial cancer [28]. Seven studies were conducted among Caucasians [21, 26, 28], and eight among Asians [22–25, 27]. All 15 studies were considered high quality; one was scored as 10, eleven as 12 and three as 13.

**Table 1:** Characteristics of studies included in the current meta-analysis

Surname	Year	Cancer type	Country	Ethnicity	Control source	Genotype method	Case				Control				MAF	HWE	Score
							AA	AC	CC	All	AA	AC	CC	All			
Garcia-Closas	2013	Breast	Multi-center	Caucasian	Mixed	Illumina array	3318	2637	557	6512	22825	15798	2828	41451	0.26	0.183	12
Liu	2013	Breast	China	Asian	PB	PCR-RFLP	733	67	0	800	686	111	3	800	0.07	0.505	13
Liu	2013	Breast	China	Asian	PB	PCR-RFLP	278	22	0	300	501	96	3	600	0.09	0.483	12
Zhou	2013	ESCC	China	Asian	PB	PCR-RFLP	501	37	2	540	478	70	2	550	0.07	0.740	13
Zhou	2013	ESCC	China	Asian	PB	PCR-RFLP	529	56	3	588	510	88	2	600	0.08	0.379	13
Fan	2014	NHL	China	Asian	PB	PCR-RFLP	187	13	0	200	346	53	1	400	0.07	0.487	12
Feng	2014	Gastric	China	Asian	HB	PCR-RFLP	208	209	51	468	210	219	64	493	0.35	0.561	10
Gansmo	2015	Breast	Norway	Caucasian	PB	LightSNiP assay	966	643	108	1717	1021	703	146	1870	0.27	0.106	12
Gansmo	2015	Colon	Norway	Caucasian	PB	LightSNiP assay	823	600	108	1531	2042	1439	266	3747	0.26	0.566	12
Gansmo	2015	Lung	Norway	Caucasian	PB	LightSNiP assay	715	515	101	1331	2042	1439	266	3747	0.26	0.566	12
Gansmo	2015	Prostate	Norway	Caucasian	PB	LightSNiP assay	1412	927	161	2500	1021	736	120	1877	0.26	0.410	12
Gao	2015	Lung	China	Asian	PB	PCR-RFLP	297	22	1	320	548	90	2	640	0.07	0.399	12
Gao	2015	Lung	China	Asian	PB	PCR-RFLP	183	17	0	200	321	77	2	400	0.10	0.248	12
Gansmo	2016	Ovarian	Norway	Caucasian	HB	LightSNiP assay	716	564	105	1385	1021	703	146	1870	0.27	0.106	12
Gansmo	2016	Endometrial	Norway	Caucasian	HB	LightSNiP assay	757	541	106	1404	1021	703	146	1870	0.27	0.106	12

MAF, minor allele frequency; HWE, Hardy-Weinberg equilibrium, ESCC, esophageal squamous cell carcinoma; NHL, non-Hodgkin lymphoma; PB, population based; HB, hospital based; PCR-RFLP, polymerase chain reaction-restriction fragment length polymorphism.

### Meta-analysis results

**Figure 2:**
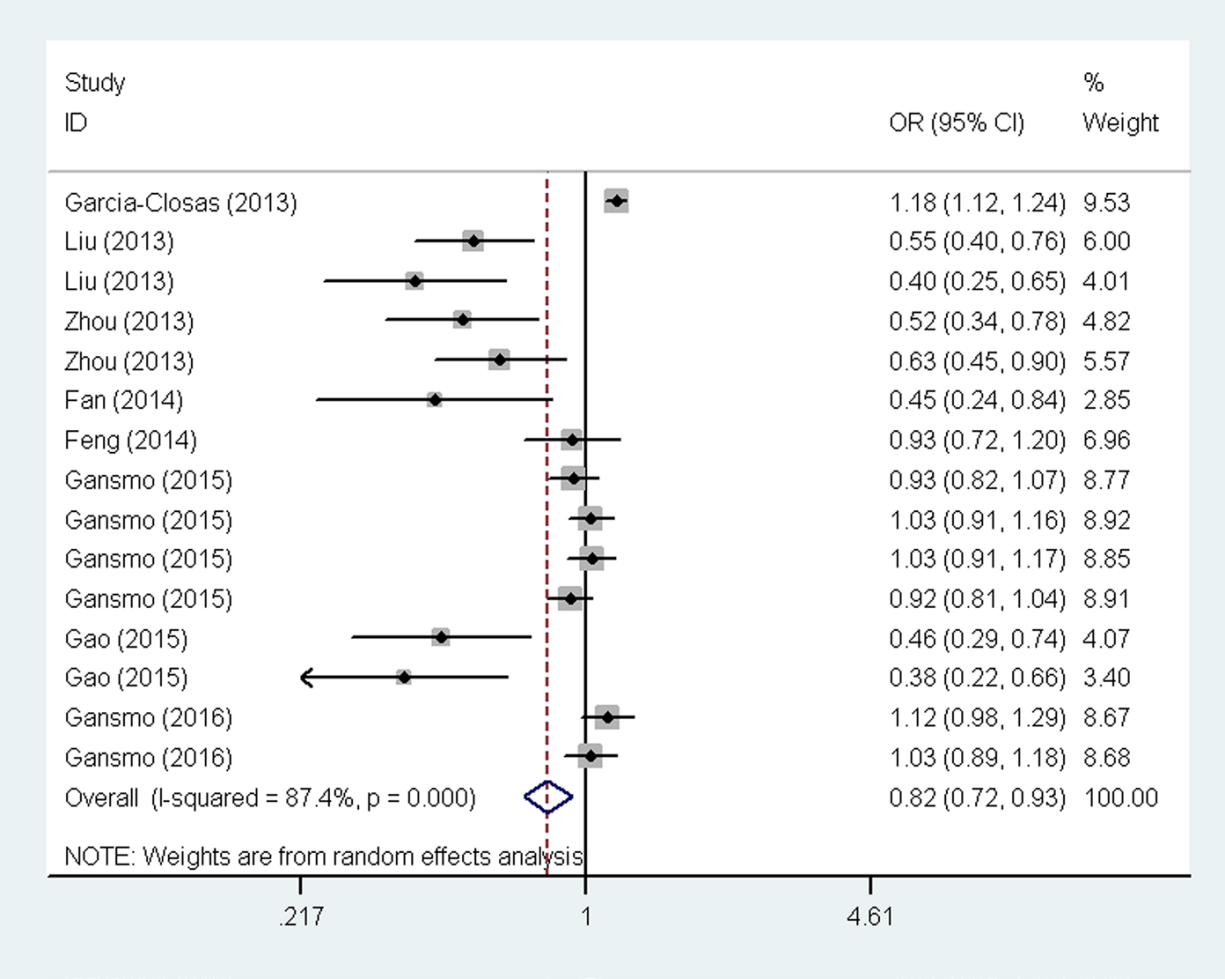
Forest plot for the association between the *MDM4* rs4245739 A > C polymorphism and cancer risk under the dominant model The horizontal lines represent the ORs and 95% CIs of single studies. The diamond represents the pooled OR and 95% CI.

The overall analysis results are shown in Figure 2 and Table 2. We found the presence of significant heterogeneity under all genetic models (*P* ^het^< 0.10); thus, we chose the random-effects model because it can generate wider confidence intervals (CIs). We found that the *MDM4* rs4245739C carriers had a significantly decreased overall cancer risk under the heterozygous [AC vs. AA: odds ratio (OR) = 0.82, 95% CI = 0.73–0.93], dominant (AC + CC vs. AA: OR = 0.82, 95% CI = 0.72–0.93), and allele contrast models (C vs. A: OR = 0.84, 95% CI = 0.76–0.94). In the subgroup analysis by ethnicity, similar results were found among Asians (AC vs. AA: OR = 0.55, 95% CI = 0.43–0.70; AC + CC vs. AA: OR = 0.54, 95% CI = 0.43–0.69; and C vs. A: OR = 0.56, 95% CI = 0.44–0.72), but not among Caucasians. When analyses were stratified by the source of controls, significant association with decreased cancer risk was found among population-based studies (AC vs. AA: OR = 0.70, 95% CI = 0.58–0.83; AC/CC vs. AA: OR = 0.70, 95% CI = 0.59–0.83; and C vs. A: OR = 0.73, 95% CI = 0.63–0.85). In the stratified analysis by quality score, significant associations were found among studies with scores ≥12 (AC vs. AA: OR = 0.81, 95% CI = 0.71–0.93; AC + CC vs. AA: OR = 0.81, 95% CI = 0.71–0.92; and C vs. A: OR = 0.84, 95% CI = 0.75–0.93).

**Table 2:** Meta-analysis of the association between *MDM4* rs4245739 A > C polymorphism and cancer risk

Variables	No. of studies	Sample size	Homozygous			Heterozygous			Recessive			Dominant			Allele		
			CC vs. AA			AC vs. AA			CC vs. AC+AA			AC+CC vs. AA			C vs. A		
			OR (95% CI)	*P* ^het^	*I*^2^ (%)	OR (95% CI)	*P* ^het^	*I*^2^ (%)	OR (95% CI)	*P* ^het^	*I*^2^ (%)	OR (95% CI)	*P* ^het^	*I*^2^ (%)	OR (95% CI)	*P* ^het^	*I*^2^ (%)
All ^a^	15	19796/49681	1.01 (0.87–1.17)	0.005	54.9	**0.82 (0.73–0.93)**	< 0.001	85.7	1.00 (0.89–1.14)	0.041	42.6	**0.82 (0.72–0.93)**	< 0.001	87.4	**0.84 (0.76–0.94)**	< 0.001	87.9
Cancer type																	
Breast	4	9329/44721	0.94 (0.55–1.61)	< 0.001	83.3	0.78 (0.57–1.06)	< 0.001	92.1	0.95 (0.59–1.51)	0.002	79.3	0.76 (0.55–1.05)	< 0.001	93.7	0.77 (0.57–1.03)	< 0.001	94.3
Lung	3	1851/4787	1.08 (0.84–1.37)	0.762	0.0	0.58 (0.29–1.19)	< 0.001	90.0	1.07 (0.84–1.35)	0.814	0.0	0.59 (0.29–1.20)	< 0.001	90.5	0.61 (0.31–1.19)	< 0.001	90.0
ESCC	2	1128/1150	1.20 (0.32–4.50)	0.759	0.0	**0.57 (0.43–0.74)**	0.484	0.0	1.27 (0.34–4.79)	0.763	0.0	**0.58 (0.45–0.76)**	0.464	0.0	**0.62 (0.48–0.79)**	0.438	0.0
Others	6	7488/4640	0.98 (0.87–1.10)	0.953	0.0	1.00 (0.89–1.11)	0.036	58.1	0.97 (0.86–1.09)	0.970	0.0	0.99 (0.89–1.10)	0.041	56.9	0.99 (0.92–1.06)	0.080	49.2
Ethnicity																	
Caucasians	7	16380/45198	1.03 (0.88–1.22)	< 0.001	75.3	1.04 (0.97–1.12)	0.012	63.1	1.02 (0.89–1.18)	0.005	67.8	1.04 (0.95–1.13)	< 0.001	75.1	1.03 (0.95–1.11)	< 0.001	80.4
Asians	8	3416/4483	0.78 (0.54–1.14)	0.908	0.0	**0.55 (0.43–0.70)**	0.007	64.0	0.81 (0.56–1.16)	0.918	0.0	**0.54 (0.43–0.69)**	0.007	64.1	**0.56 (0.44–0.72)**	0.001	71.2
Source of control																	
PB	11	10027/7737	0.96 (0.85–1.08)	0.763	0.763	**0.70 (0.58–0.83)**	< 0.001	83.4	0.96 (0.85–1.09)	0.807	0.0	**0.70 (0.59–0.83)**	< 0.001	84.0	**0.73 (0.63–0.85)**	< 0.001	84.3
HB	3	3257/2363	0.97 (0.81–1.15)	0.623	0.623	1.07 (0.97–1.18)	0.461	0.0	0.94 (0.79–1.11)	0.757	0.0	1.05 (0.96–1.16)	0.387	0.0	1.02 (0.95–1.10)	0.371	0.0
Mixed	1	6512/41451	**1.36 (1.23–1.49)**	/	/	**1.15 (1.09–1.21)**	/	/	**1.28 (1.16–1.41)**	/	/	**1.18 (1.12–1.24)**	/	/	**1.16 (1.11–1.21)**	/	/
Quality score																	
≥ 12	14	19596/49281	1.03 (0.88–1.19)	0.009	53.9	**0.81 (0.71–0.93)**	< 0.001	86.6	1.02 (0.90–1.16)	0.055	41.0	0.81 (0.71–0.92)	< 0.001	88.2	**0.84 (0.75–0.93)**	< 0.001	88.5
< 12	1	200/400	0.81 (0.53–1.22)	/	/	0.96 (0.74–1.26)	/	/	0.82 (0.55–1.21)	/	/	0.93 (0.72–1.20)	/	/	0.92 (0.76–1.11)	/	/

Het, heterogeneity; ESCC, esophageal squamous cell carcinoma; HB, hospital based; PB, population based.

### The *MDM4* mRNA expression by genotypes

**Figure 3:**
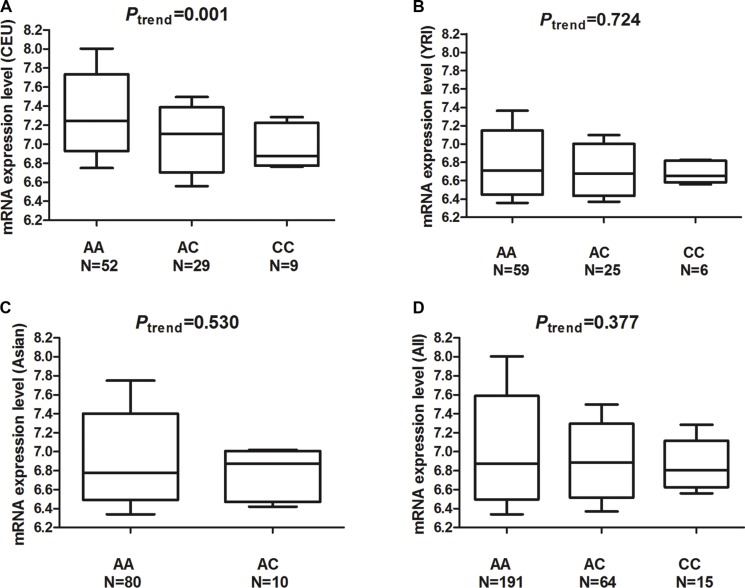
mRNA expression level of the *MDM4* gene in Epstein-Barr virus (EBV)-transformed lymphoblastoid cell lines (**A**) mRNA expression in 90 cell lines from unrelated CEU (Utah residents with ancestry from northern and western Europe) individuals. (**B**) mRNA expression in 90 cell lines of unrelated YRI (Yoruba in Ibadan, Nigeria) individuals. (**C**) mRNA expression in 90 cell lines of unrelated Asian individuals. (**D**) mRNA expression in 270 cell lines of all individuals.

In the genotype-based mRNA expression analysis (Table 3 and Figure 3) using public datasets, we found the rs4245739C allele carriers had trends toward decreased mRNA expression level among Caucasians, Asians, Africans, and all subjects. The decrease in the *MDM4* mRNA expression reached a statistical significance among the Caucasians (AC vs. AA: *P* = 0.002; CC vs. AA: *P* = 0.004, and AC + CC vs. AA: *P* = 0.0002), but not among other populations.

**Table 3:** *MDM4* mRNA expression by the genotypes of rs4245739 A > C, using data from the HapMap^a^

Population	genotypes	No.	Mean ± SD	*P*^b^	*P*_trend_^c^
CEU	AA	52	7.28 ± 0.31		0.001
	AC	29	7.06 ± 0.27	0.002	
	CC	9	6.96 ± 0.20	0.004	
	Dominant	38	7.03 ± 0.26	0.0002	
YRI	AA	59	6.75 ± 0.23		0.724
	AC	25	6.72 ± 0.22	0.578	
	CC	6	6.68 ± 0.11	0.271	
	Dominant	31	6.71 ± 0.20	0.459	
Asian	AA	80	6.86 ± 0.31		0.530
	AC	10	6.79 ± 0.23	0.530	
	CC	0	/	/	
	Dominant	10	6.79 ± 0.23	0.530	
All	AA	191	6.94 ± 0.36		0.377
	AC	64	6.88 ± 0.29	0.271	
	CC	15	6.85 ± 0.22	0.165	
	Dominant	79	6.88 ± 0.28	0.135	

CEU, 90 Utah residents with ancestry from northern and western Europe; YRI, 90 Yoruba in Ibadan, Nigeria.

a Genotype data and mRNA expression levels for *MDM4* by genotypes were obtained from the HapMap phase II release 23 data from EBV-transformed lymphoblastoid cell lines from 270 individuals.

b Two-sided Student's *t* test within the stratum.

c *P* values for the trend test of *MDM4* mRNA expression among 3 genotypes for each SNP from a general linear model.

### Sensitivity analysis and publication bias

We conducted sensitivity analysis to assess the influence of each individual study on the pooled ORs and 95% CIs by omitting one study each time. No individual study could alter the pooled ORs significantly, which demonstrated that the studies were relatively statistically robust. Additionally, we found that no single study could alter the publication bias in an obvious manner (data not shown).

## DISCUSSION

In the present meta-analysis comprising 69477 subjects from 15 studies, we completed the first comprehensive evaluation of the association between *MDM4* gene rs4245739 A > C polymorphism and overall cancer risk. The pooled results indicate that the *MDM4* rs4245739 A > C polymorphism was significantly associated with decreased overall cancer risk, which was consistent with the results of our genotype-based mRNA expression analysis.

SNPs are the most common type of genetic variations. The majority of SNPs are silent or have limited influence on the function and expression of genes. Only a small fraction of SNPs have been reported to be potentially functional and associated with cancer susceptibility [29–32], in accordance with the theory of the driver and passenger somatic mutations in human cancer genome [33]. The influence of genetic variations, particularly SNPs, on an individual's cancer susceptibility under similar environmental exposures, has been widely investigated and has become a hot research topic worldwide [34]. The association between SNPs and cancer risk may be strongly cancer-specific [35]. Numerous previous studies have investigated the association between *MDM4* gene polymorphisms and cancer susceptibility [21–28].

The *MDM4* gene (also known as *HDMX* or *MDMX*) is located at chromosome 1q32, a region that is frequently found to be amplified in cancer [36]. This gene contains 11 exons and encodes a protein of 490 amino acids [37], in which at least 2709 SNPs have been identified (http://www.ncbi.nlm.nih.gov/projects/SNP). Among these SNPs, a potentially functional polymorphism (rs4245739 A > C) has received a great deal of recent attention. The rs4245739 A > C polymorphism was first identified in 40 German patients with familial breast cancer through sequencing the whole coding and flanking untranslated regions of *MDM4* gene [38]. This polymorphism, located in the 3′ UTR of *MDM4* gene, generates a miR-191 target binding site. As a result, miR-191 selectively binds to mRNA harboring the *MDM4* rs4245739C allele to decrease the expression of *MDM4* gene at both mRNA and protein levels, but not mRNA with *MDM4* rs4245739A allele (wild-type). The decreased MDM4 expression caused by miR-191 binding might increase the activity of p53 and consequentially modify an *MDM4* rs4245739A allele carriers' susceptibility to ovarian cancer and retinoblastoma [39, 40]. Additionally, the *MDM4* rs4245739 AC genotype may be associated with increased overall survival in non-small cell lung cancer, when compared to the AA genotype [41]. Despite the biological plausibility, studies investigating the association between this polymorphism and cancer risk have yielded inconclusive results [21–28]. For instance, some studies found that the rs4245739 A > C polymorphism was significantly associated with decreased cancer risk [22–24, 27]; in contrast, Garcia-Closas et al. [21]. reported that this polymorphism was associated with increased breast cancer risk. Moreover, others found that this polymorphism may have weak or no effect on cancer risk [25, 26, 28]. It is widely recognized that different cancer types have unique characteristics and involve differing signal pathways. Even among the same cancer type, cancers from different patients display significant heterogeneity. The possible reasons for discrepancies regarding cancer susceptibility may be ascribed to tumor specificity, differences in ethnicity, and variations in sample sizes included in each investigation. When we combined all available investigations, we found that the rs4245739C allele carriers had decreased cancer risk, especially among Asians. Moreover, we also found that the rs4245739C allele was associated with decreased mRNA expression of *MDM4* by genotype-based mRNA expression analysis, which could provide further biological evidence of the possible mechanism of this polymorphism.

Although this is the first meta-analysis investigating the association between *MDM4* gene rs4245739 A > C polymorphism and overall cancer risk, some limitations should be discussed. First, although all of the eligible studies were pooled together, the total number of studies and the sample sizes for most types of cancer were still relatively small. As a result, statistical power might be limited while evaluating the association of interest, especially in the subgroup analysis. For instance, there was only one study available for several types of cancer including non-Hodgkin lymphoma, gastric cancer, colon cancer, and prostate cancer. No pooled study could be performed for these cancers. Second, there was heterogeneity among the included studies, which might stem from the inconsistent results derived from different cancers and ethnicities. Third, nearly all of the studies included in this meta-analysis were conducted among Asians and Caucasians. In light of genetic and geographical differences, more investigations from different areas and ethnicities are required to verify our findings. Fourth, the lack of original data limited the further evaluation of potential gene-gene and gene-environment interactions that may modulate cancer risk. Fifth, we may have missed some publications, especially studies without genotype data and those with negative results that were not published. For example, the genotype data for the control subjects were not available in the investigation carried out by Wynendaele et al. [39]. As a result, this study was not included in the current meta-analysis. Finally, publication bias may exist since only published studies were included in our meta-analysis. So, the conclusions drawn from the current study should be interpreted with caution.

In conclusion, our meta-analysis revealed that *MDM4* gene rs4245739 A > C polymorphism was associated with a reduction in overall cancer susceptibility. Due to the limitations of the current meta-analysis, future studies with larger sample size and different ethnicities and cancer types are needed to confirm these findings.

## MATERIALS AND METHODS

### Publication search

We conducted a comprehensive literature search for all relevant publications concerning the association between *MDM4* rs4245739 A > C polymorphism and cancer risk from MEDLINE, EMBASE, and CBM database (prior to 5 September, 2016). The following search terms were used: “*MDM4* or *HDMX* or *MDMX* or *MRP1* or rs4245739”, “cancer or carcinoma or tumor or neoplasm”, and “polymorphism or variant or variation”. We also searched for additional relevant studies from the references of retrieved publications.

### Inclusion and exclusion criteria

Studies meeting the following inclusion criteria were included: (1) evaluated the association between *MDM4* rs4245739 A > C polymorphism and cancer risk; (2) case-control study or cohort study; (3) genotype distributions were available for both cases and controls; (4) published in English or Chinese.

Exclusion criteria included: (1) not case-control study design; (2) did not evaluate the association between *MDM4* rs4245739 A > C polymorphism and cancer risk; (3) studies with overlapping participants; (4) conference abstracts, review articles, comments, meta-analyses, or editorial articles. In the case of duplicate or overlapping studies, only the most complete one was included.

### Data extraction

Two authors (Chaoyi Xu and Jinhong Zhu) independently extracted the following information from each investigation: the first author's surname, publication year, country, ethnicity, control source, genotyping method, as well as number of case and control with AA, AC and CC genotypes. All disagreements were resolved through discussion between these two investigators until a consensus was reached.

### Genotype-based mRNA expression analysis

We performed genotype-based mRNA expression analysis as we described previously [3, 42–45]. Genotypes data of *MDM4* rs4245739 A > C polymorphism for 270 individuals with three ethnicities were obtained from HapMap (http://www.hapmap.org). The *MDM4* gene mRNA expression data for the same 270 individuals were downloaded from SNPexp (http://app3.titan.uio.no/biotools/tool.php?app=snpexp).

### Statistical analysis

The strength of the association between *MDM4* rs4245739 A > C polymorphism and overall cancer risk were assessed using crude OR and 95% CI under the homozygous (CC vs. AA), heterozygous (AC vs. AA), recessive (CC vs. AC + AA), dominant (AC + CC vs. AA), and allele contrast models (C vs. A). Goodness-of-fit χ^2^ test was adopted to test deviation from HWE for the genotypes of control subjects. Heterogeneity was assessed using χ^2^-based *Q* test, and was considered as significant when *P* < 0.10. We also qualified the heterogeneity using *I*^2^ statistics, a value with a range from 0% to 100%. A higher *I*^2^ value indicates a greater degree of heterogeneity [46]. When significant heterogeneity was found, random-effects model [47] would be adopted; otherwise, fixed-effects model (the Mantel-Haenszel method) [48] would be used. The quality of each investigation was evaluated by quality assessment criteria (Supplementary Table S1) as we described previously [43]. Subgroup analysis was conducted by cancer type (investigations with only one study would be merged into the “others” group), ethnicity, source of control and quality score of investigations. Sensitivity analysis was conducted to evaluate the stability of the results. The pooled ORs and 95% CIs were estimated by excluding one study at a time to evaluate the influence of single investigation. The potential publication bias was estimated using Begg's funnel plot [49] and Egger's linear regression test [50]. In terms of genotype-based mRNA expression, two-sided Student's *t* test was used for the comparison of two groups, and one-way ANOVA was adopted for comparison among three different genotypes. The statistical analysis was performed with STATA software (version 11.0; Stata Corporation, College Station, TX). All the statistics were two-sided, with *P* < 0.05 indicating statistical significance.

## SUPPLEMENTARY MATERIALS TABLES


